# Aligning functional network constraint to evolutionary outcomes

**DOI:** 10.1186/s12862-020-01613-8

**Published:** 2020-05-24

**Authors:** Katharina C. Wollenberg Valero

**Affiliations:** Department of Biological and Marine Sciences, University of Hull, Cottingham Road, Kingston-Upon-Hull, HU6 7RX UK

**Keywords:** Evolution, Constraint, Adaptation, Systems biology

## Abstract

**Background:**

Functional constraint through genomic architecture is suggested to be an important dimension of genome evolution, but quantitative evidence for this idea is rare. In this contribution, existing evidence and discussions on genomic architecture as constraint for convergent evolution, rapid adaptation, and genic adaptation are summarized into alternative, testable hypotheses. Network architecture statistics from protein-protein interaction networks are then used to calculate differences in evolutionary outcomes on the example of genomic evolution in yeast, and the results are used to evaluate statistical support for these longstanding hypotheses.

**Results:**

A discriminant function analysis lent statistical support to classifying the yeast interactome into hub, intermediate and peripheral nodes based on network neighborhood connectivity, betweenness centrality, and average shortest path length. Quantitative support for the existence of genomic architecture as a mechanistic basis for evolutionary constraint is then revealed through utilizing these statistical parameters of the protein-protein interaction network in combination with estimators of protein evolution.

**Conclusions:**

As functional genetic networks are becoming increasingly available, it will now be possible to evaluate functional genetic network constraint against variables describing complex phenotypes and environments, for better understanding of commonly observed deterministic patterns of evolution in non-model organisms. The hypothesis framework and methodological approach outlined herein may help to quantify the extrinsic versus intrinsic dimensions of evolutionary constraint, and result in a better understanding of how fast, effectively, or deterministically organisms adapt.

## Background

### Genetic constraint and evolutionary outcomes

Understanding the genetic dimension of evolutionary constraint is crucial to understanding adaptation and other repeatedly observed outcomes of evolution such as convergent phenotypes, rapid adaptation, or genic evolution (see Glossary in Table 1). For example, divergent genetic populations of the well-studied Caribbean lizard *Anolis cybotes* [3] have nonetheless evolved convergent phenotypic, ecological, reproductive, and physiological adaptations to high elevations on three separate mountain chains, which is mirrored by genomic adaptations in a subset of genes [4–7]. These observations made in natural populations suggest that the variants available to mutation and selection may be constrained at the genomic level, enabling faster adaptations and higher rates of convergent evolution than were possible without constraint.

**Table 1:** Glossary of terms

**Evolutionary constraint**	[1]: the phenomenon of evolution producing a finite number of genomic and associated phenotypic outcomes from a near infinite number of possible genetic variants.
**Genetic constraint**	The portion of evolutionary constraint, which is determined at the level of genes or their gene products, for example codon constraint or developmental genetic pathways.
**Functional network constraint**	The portion of network constraint attributed to the structure or architecture of gene interactions that can be expressed in the form of a network. Networks consist of nodes (genes) and edges (functional interactions between these genes).
**Genic evolution**	The phenomenon of different evolutionary outcomes being the outcome of independent mutation and selection events in different genes. For example, the occurrence of convergent evolution in diverging populations, both of which are caused by evolution in distinct genes.
**Rapid adaptation**	The phenomenon of adaptive change in allele frequencies of a population to natural selection, taking place within just a few generations.
**Convergent evolution / convergence**	Traditionally defined as similar phenotypes evolving from similar selective pressure in response to similar environments [2]. May be caused at the genomic level through genomic re-use of the same genes or alleles, which is also called parallel genetic evolution or genomic re-use.
**Gene dispensability**	A variable to estimate gene essentiality. The less dispensable a gene is for organismal growth and function, the more essential it is. An estimator for the mean fitness effect of all possible mutations of a gene across environments the cell is likely to encounter. In yeast, this is experimentally determined through knockouts.
**Pleiotropy and cost of complexity**	Traditionally defined as one gene influencing more than one trait. In the papers cited in this study, has been defined as gene products with more than one functional interactions with other gene products, with the link to pleiotropy of phenotypic traits being implied. It is therefore called “gene pleiotropy”.
**Gene expression level CAI**	The amount of mRNA produced by each gene in regular somatic cells. CAI (Codon Adaptation Index) is used as a substitute variable in this paper, and is derived from codon use bias in yeast that correlates with mRNA levels.
**Omega ω**	The ratio of nonsynonymous to synonymous substitutions dN/dS. It is assumed that dS remains constant, and dN is used here as an estimator for directional evolution.
**Gamma γ**	A score developed for estimating events of rewiring functional connections between network nodes over the course of evolution. Developed on the example of five species of yeasts.
**Neighborhood connectivity**	A network statistic used to describe the structure of a functional genetic network. Describes the number of connections of all neighbors of each node. Highest values are expected in intermediately located nodes within a network.
**Betweenness centrality**	A network statistic used to describe the structure of a functional genetic network, describing where a node lies within paths between other nodes. Nodes with many paths progressing through them may be important in transmitting information. Highest values are expected in nodes central to a network.
**Average shortest path length**	A network statistic used to describe the structure of a functional genetic network. Shortest distance between a node and other nodes. Highest values are expected in peripheral nodes of a network.

**Fig. 1:**
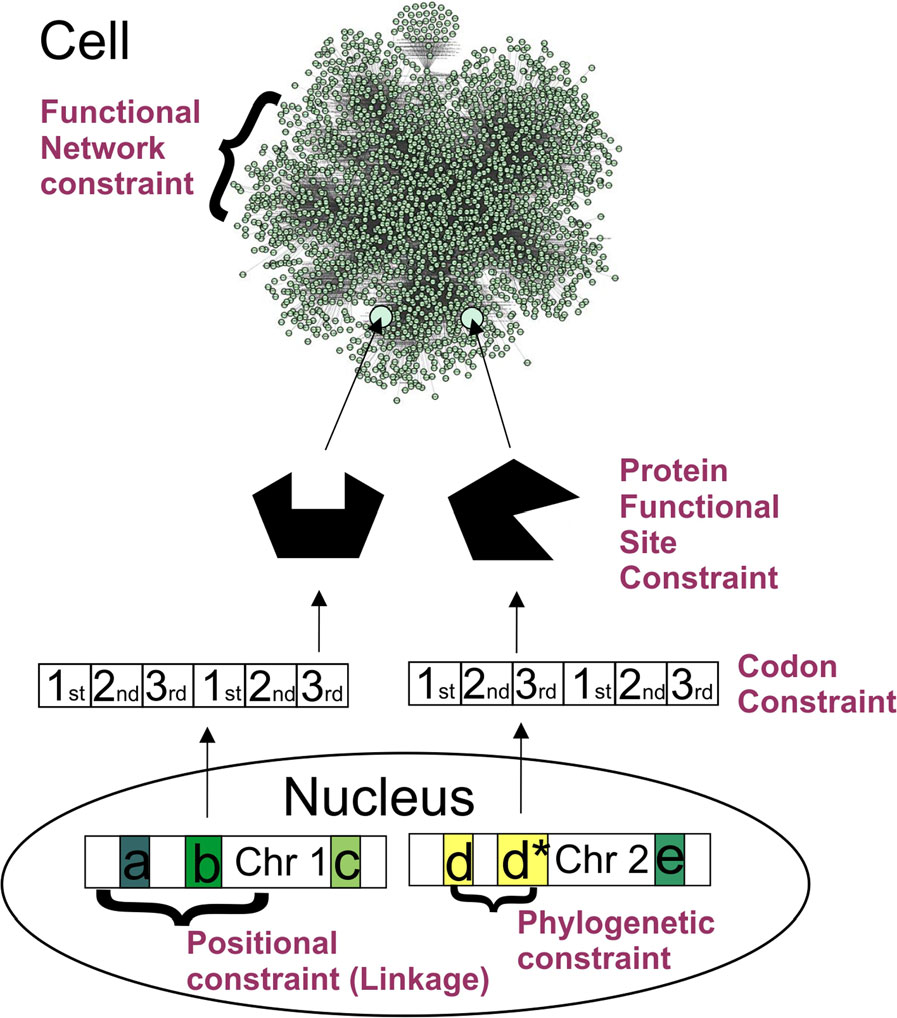
Examples for different levels of genetic constraint. Linkage is a transient constraint, which is broken up through recombination or other chromosome rearrangements. If a gene arises through duplication, phylogenetic constraint means that the function of its gene product may be non-independent with relation to the ancestral gene product. Codon constraint describes the likelihood of the different codon positions to produce beneficial mutations. Protein functional site constraint describes constraint located in genomic regions that code for functional sites of proteins versus other regions of the proteins. This is related to the idea that gene products form a functional genomic network. Within this network, interactions of these gene products also pose an element of constraint on evolution, but this is not well researched

Many studies have shown the non-independence of genes from one another, be it through physical linkage, phylogenetic relationship (e.g., in the case of whole genome duplications), or functional interaction (Fig. 1). Futuyma [8] cited Schluter [9], noting that correlations between genes could reduce the degrees of freedom on which selection can operate. Mayr [10] stated that “coadapted” genes are a result of natural selection, being brought together to form a “balanced system”, but ruled out that such gene complexes would be of any interest to evolutionary biology, as ultimately only the complete phenotype is selected (8, p.184ff). Nonetheless, evolutionary trajectories of complex phenotypes have been extensively studied through the concept of the genetic variance-covariance or G-matrix [11]. Some mechanistic properties of the genome leading to the constraints that can be expressed as a G-matrix are trait polygeny, trait pleiotropy, and linkage (Figs. 1), [12], but the evolutionary constraints of correlated traits implied from a G-matrix can be rapidly overcome in only a few generations [13], hinting at additional genomic properties influencing evolutionary constraint. A more detailed look into the mechanistic basis of constrained phenotypic evolution at the molecular level is therefore necessary, and now made possible through the rapid accumulation of genomic and other molecular -omics data sets in the public domain.

### Genetic constraint through gene functional importance

The functional importance of a gene has long been thought to cause such evolutionary constraint at the molecular level: protein-coding genes that are indispensable for the organism should be highly conserved and thus, be constrained through evolution, as most nonsynonymous mutations would be detrimental to protein function and thus would most likely result in non-adaptive phenotypes. Consequently, these genes should have a lower rate of molecular evolution. Such genes have formerly been identified through their “dispensability”. This term describes how essential genes are for organismal function within a certain environmental context, which can be estimated through knockout experiments (Table 1).

Zhang and Yang [14] reviewed evidence from empirical studies, but found that essential genes are not evolving more slowly than nonessential genes. Instead, highly expressed genes seem to have lower rates of protein evolution (dubbed the “E-R anticorrelation” [15, 16], which some authors relate to translational selection on amino acids with different metabolic cost [15]. Many studies have ascribed an important role to gene expression levels in constraining the evolutionary rate of proteins [17–19]. But perhaps, functional importance needs to be defined differently than via gene essentiality or dispensability, and expression level may be a correlative variable linked to another cause. In *Saccharomyces cerevisiae* (in following: yeast), which was used for many studies on protein evolutionary rate and functional importance, essential genes are required for organismal growth and performance under optimal environmental conditions. A gene that renders an organism nonfunctional may thus predominantly be active in genetic pathways related to development and growth. However, in a multicellular organism such as a vertebrate, also the genes that are essential for organismal viability and reproduction are of high functional importance, and potentially could be under evolutionary constraint [20], such as in the example of genes coding for eye color determining mating success in *Drosophila melanogaster* [21]. A high proportion of the human genome has also been found to be under selective constraint in other mammals, indicating that gene dispensability is not a binary variable [22]. As reviewed by Zhang and Yang [14], Wilson et al. [23] suggested that evolutionary rate may be determined by both functional importance and functional constraint [14, 23]. If functional importance measured as (negative) gene dispensability does not predict variations and constraints of evolutionary rate, perhaps functionally more constrained genes are the ones evolving slowly. Prior studies have attempted to identify functional constraint in terms of which sites within a protein are essential for performing its function, called protein functional site constraint in Fig. 1. The Neutral Theory [24] already identified codon constraint where nonsynonymous mutations are of larger consequence than synonymous ones as being important for evolution (Fig. 1).

### Genetic constraint through gene pleiotropy and network architecture

During the recent decades, network thinking has emerged as a powerful approach for better understanding biological realities [25]. The network concept might also have deep implications in evolutionary biology. Gene interaction networks were found to evolve either faster or slower than comparable genes functioning without being connected to others [26–29], and gene regulatory circuits convergently evolve in the absence of shared ancestry [30]. The overall network architecture or hierarchy of genes within the network is likely to contribute to the speed and mode of evolution and the phenotype components associated with it, regulated through functional constraint of nodes within the network [25]. For example, a study by Jeong and colleagues [31] found that genes with many functional interaction partners are also likely to be essential, which, however, does not provide enough evidence to extrapolate directly from functional constraint to evolutionary outcome.

Functional genetic network structure has been shown to affect evolutionary outcomes through “gene pleiotropy” in yeast: gene products that interact with many others are thought to be involved in many cellular pathways and by that means, to have multiple (pleiotropic) effects on the cellular function [32, 33]. Fitness effects of mutations in pleiotropic genes could be partitioned across several phenotypic components, increasing the likelihood of maladaptive effects, which means that they should be more conserved through evolution and evolve more slowly [32]. However, it is important to note, despite that a connection between pleiotropic gene and pleiotropic phenotype was implied in these studies [32, 33], gene pleiotropy or the number of functional connections a gene has with others should be regarded as a distinct concept from phenotypic pleiotropy, unless such a relationship to pleiotropic phenotype has been demonstrated. Proteins with many interactants may be constrained in the evolution of their functional sites to instances of co-evolution with the interactant genes, in order to maintain their functionality [34]. A corresponding model of evolutionary constraint on evolution through gene pleiotropy that was explicitly based on functional network node hierarchy within an interactome was proposed by Pavlicev and Wagner [35]. They argued that for genetic adaptation in a target gene to happen, selection has to overcome the inertia generated through stabilizing selection of the genes functionally connected with the target [35]. The premise of this model is that any change in genotype-phenotype interaction represents a change in a developmental pathway and, due the position of a gene within a network, will have pleiotropic effects on the phenotype [35]. Empirical research into this topic found that most pleiotropic genes with many interaction partners only had a small pleiotropic effect on the phenotype, but some genes with large phenotypic effect were also more pleiotropic [20]. High gene pleiotropy is assumed to have a cost for adaptation, which was explained as nodes central to a network with many interaction partners evolving slower [20, 36]. This idea, dubbed the “cost of complexity” [37] would lead to faster evolution of organisms with less complex genomic architecture due to this constraint being relaxed [20], and to adaptive selection on standing genetic variation preferentially to occur in genes with low pleiotropic effects [38]. Concerning evolutionary outcomes, gene pleiotropy was suggested to limit events of genomic co-evolution [34], genomic adaptation [38], and convergent evolution [38] in nodes central to a network. Consequently, the properties of nodes within a functional genetic network may be informative to understand their evolutionary constraint. However, gene pleiotropy was defined by most of these authors [32–35, 37] as synonymous with the number of interactants, and also with centrality in a network - but looking at more recently generated interactomes, nodes topologically central to the network are usually not the nodes with the highest number of connections. Instead, nodes with most connections are located in intermediate positions within a network [39]. The number of edges of a node consequently, may not be sufficient to disentangle the effects of network structure on evolutionary constraint since it only measures one of a network’s many properties. The concept of variable genomic networks existing within populations was first explored by Wagner [40] and was represented through a hypothetical “genotype space” of similar phenotypes that might correspond to the concept of “phenotypic optima”. Selection can cause a population to modify their genotype networks in a way that renders them more robust to changes in the fitness landscape.

While the concept of network architecture influencing evolutionary outcomes is known from the studies outlined above and from others, in many cases this concept has not been sufficiently transformed into testable hypotheses yet, and correspondingly, no straightforward methodology exists for biologists to test them empirically. **The first aim** of this paper is to deconstruct the abstract concept of gene pleiotropy by setting genomic network architecture in relation to the three evolutionary outcomes: 1) genomic re-use generating convergent phenotypes, 2) the simultaneous occurrence of convergence and divergence within a genome, resulting in genic adaptation, and 3) the speed of adaptation. For this purpose, I propose three categories of nodes with different putative evolutionary trajectories. I set these categories in relation to previously defined hypotheses and expectations aligning functional network constraint to evolutionary outcomes. **The second aim** is to demonstrate how these hypotheses can be quantitatively tested. First, the nodes of the yeast interaction network are transformed into categories based on network statistical parameters and discriminant function analysis. Secondly, these are then tested for differences of estimators of evolutionary outcomes using data from yeast evolution. For this I use published data, which aligns this study to others with similar questions [15, 41, 42] but utilizes a novel approach.

## Results

### Aim 1: novel node classification scheme based on network statistics

**Fig. 2:**
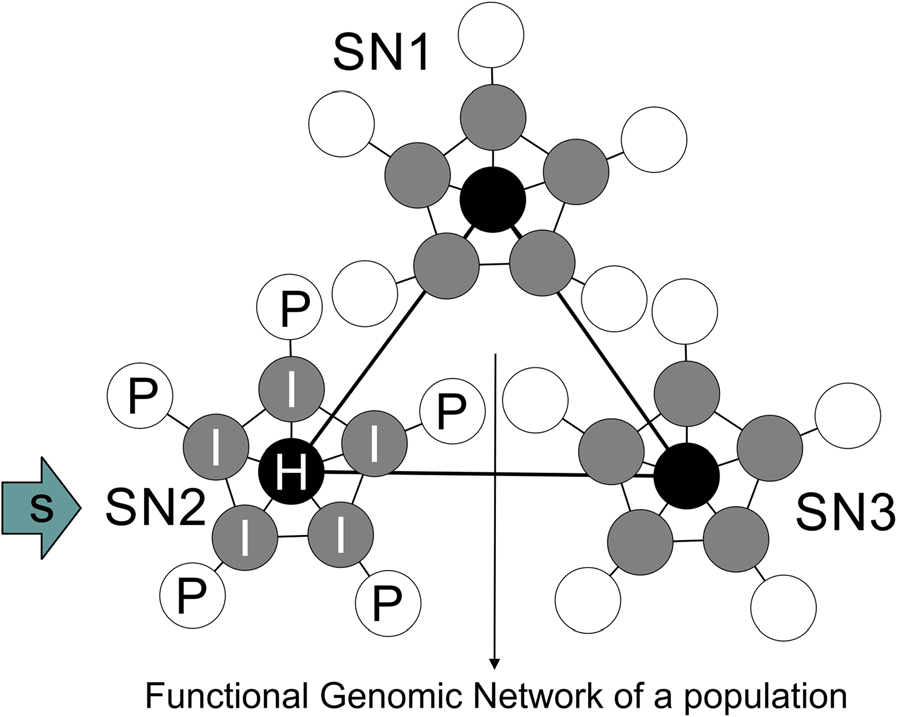
Proposed testable relationship between functional genomic network architecture, network node position, and evolutionary outcomes. SN are subnetworks within the functional genomic network of a population with distinct functions (e.g., metabolic pathways). Standing genetic variation exists within nodes, but depends on their position within the network. Black nodes (H) are essential for organismal function and not likely to accumulate non-synonymous mutations; Grey nodes (I) are functionally connected with many others and constrained in accumulating non-synonymous mutations. White nodes (P) are functionally connected to fewest others and most likely to accumulate non-synonymous mutations. Resulting from this, three evolutionary outcomes can be explained: Rapid adaptation is facilitated in white nodes through their high standing genetic variation. Selection being constrained to operate on these nodes in a specific subnetwork increases the speed of adaptation. Convergent evolution is facilitated through the finite number of networks that are related to specific functions and shared among species through common ancestry. The likelihood of convergent evolution within one subnetwork in response to selection increases through the moderate level of genetic variance, combined with constraint posed by the high number of connections to other nodes. Genic evolution is facilitated through the selection pressure only having an effect in the subnetwork with organismal functions related to it but not in others. Selection is likely to operate on standing genetic variation, which is likely concentrated in white nodes (shown as blue squares). These different processes can explain the coexistence of convergent and divergent (rapid, genic) evolution within the genomes of a population

**Fig. 3:**
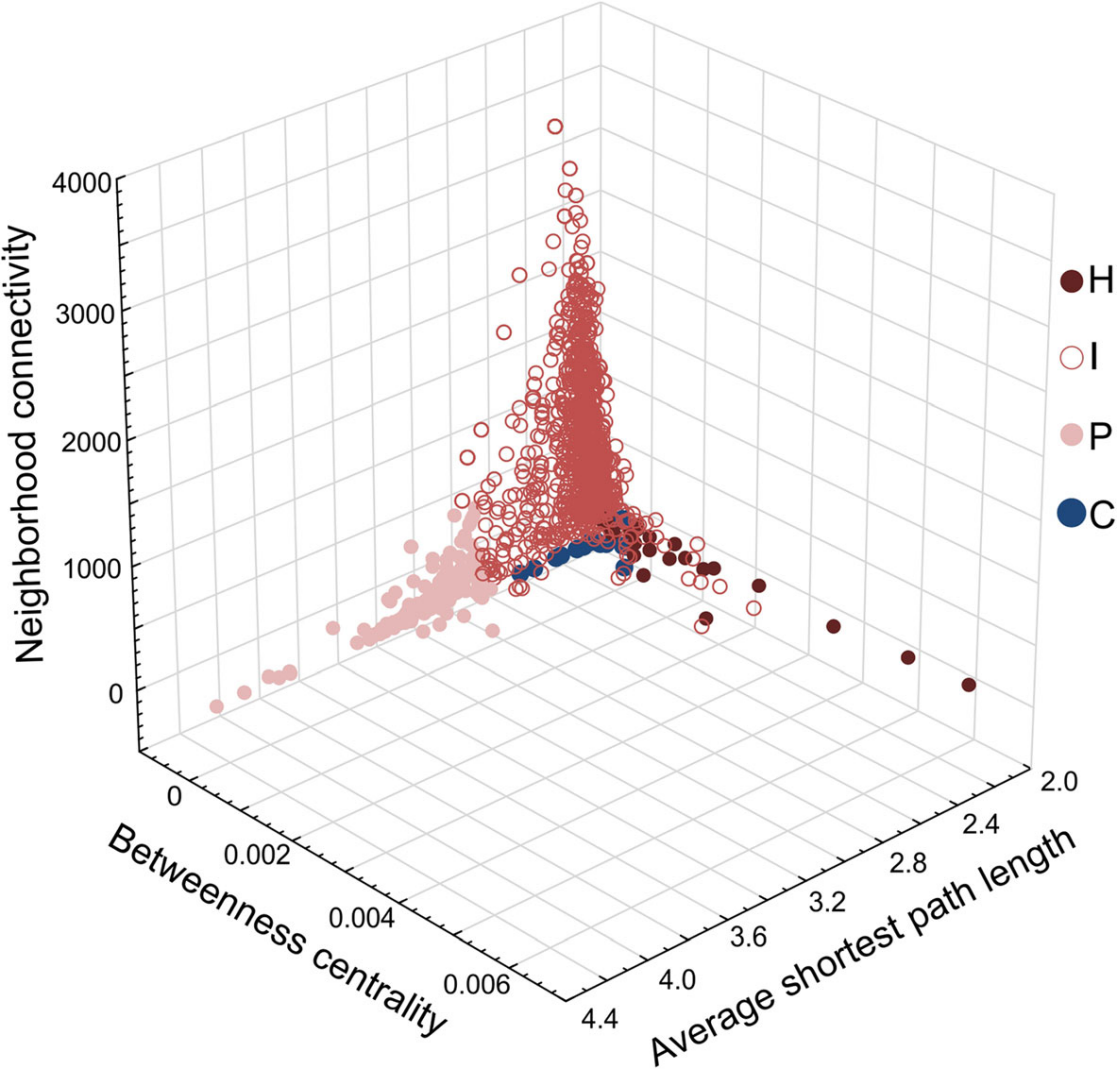
Distribution of yeast interactome nodes within network parameter space (neighborhood connectivity, average shortest path length, and betweenness centrality). The top values for each axis are colored in shades of red (light, filled: P-nodes; light, open: I-nodes; dark, filled: H-nodes). Convergent evolution nodes are indicated in dark blue. These top values for each axis formed the basis to classify the remaining nodes based on discriminant function analysis

**Fig. 4:**
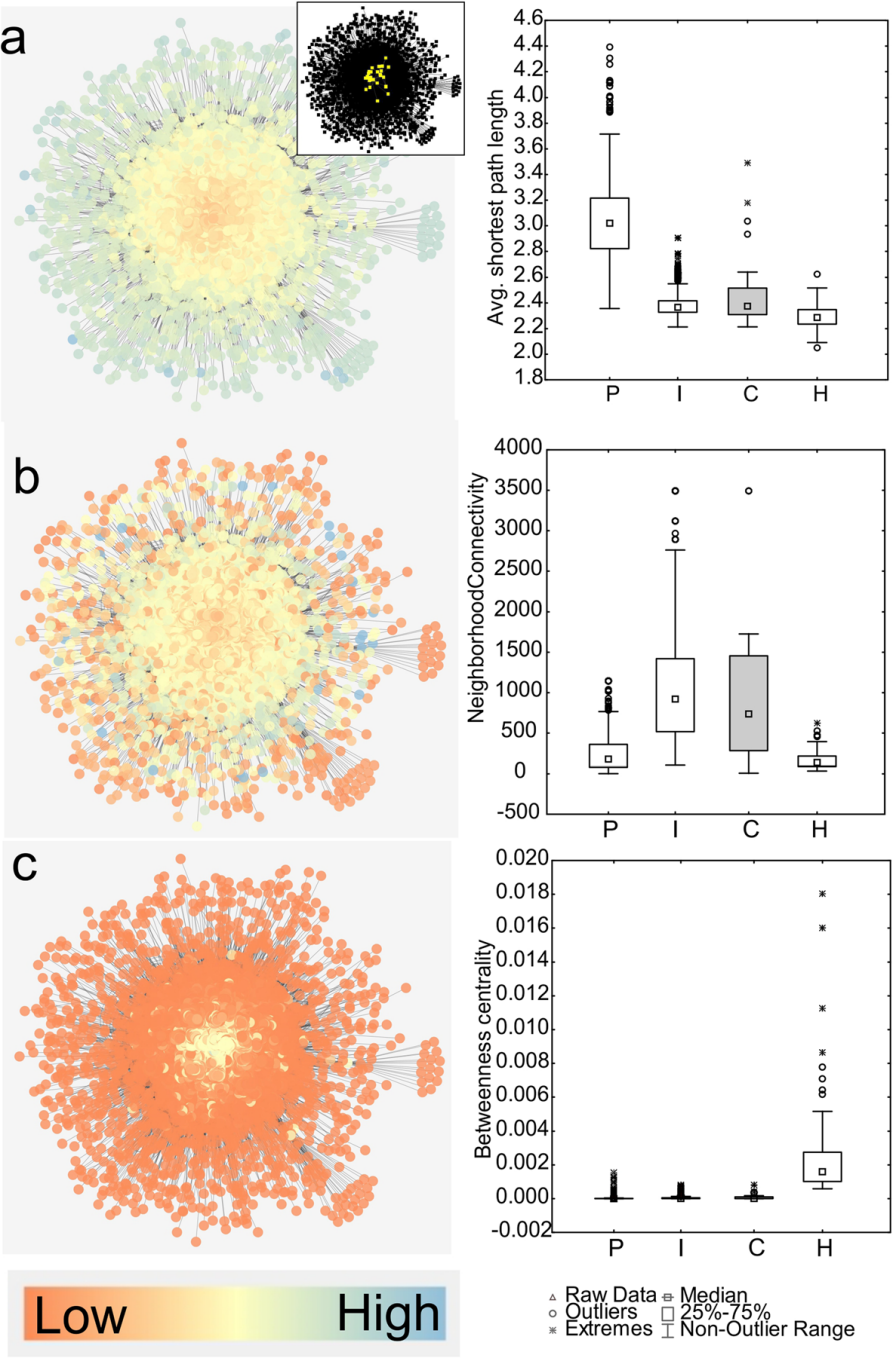
Visualization of node classification scheme in yeast interactome. Values of **a**) average shortest path length, **b**) neighborhood connectivity, and **c**) betweenness centrality within the yeast interactome (left panels), and values for the DFA-derived hierarchical node categories P, I and H, and for nodes known to be under convergent evolution in yeasts (C, *N* = 18). The small inset network shows the location of convergently evolved genes (C-nodes) within the interactome (yellow nodes)

A testable, hypothetical scenario of how functional genetic network architecture could influence evolutionary outcomes is shown in Fig. 2. The relationship between pre-existing hypotheses and results from the present study is shown in Table 2. Values for three network statistical parameters were obtained from the yeast interactome whose definition corresponds to the above outlined node types. Those parameters were average shortest path length (maximal in peripheral nodes), neighborhood connectivity (maximal in nodes intermediate to the network), and betweenness centrality (maximal in nodes connecting subnetworks). Maximal values for each statistical parameter were used to bin nodes into P, I and H nodes (which stands for peripheral, intermediate, and hub nodes). A Discriminant Function Analysis yielded significant support for the allocation of network statistical parameters to these P, I, and H node categories (Fig. 3, Table 3). To explore the network position of nodes that have undergone convergent adaptation, yeast ORF IDs that were demonstrated experimentally to show convergent genomic adaptation in independent experiments, strains, or species of yeasts were identified from the literature ([38, 48–52], Table 4). These nodes were classified as C-nodes. All network statistical parameters significantly differed between node categories, as shown with Kruskal-Wallis tests: Average shortest path length: KW-H (3,2208) = 1220.590, *p* < 0.0001; neighborhood connectivity, KW-H(3,2204) = 926.571, *p* < 0.0001; betweenness centrality KW-H(3,2208) = 293.849, *p* < 0.0001 (Fig. 4). I-nodes contain the highest number of edges and connect sub-networks, but are not defined with respect to their expression. C-nodes had network statistical parameters most similar to I-nodes (cf. inset network in Fig. 4).

**Table 2:** Hypotheses relating network constraint to evolutionary outcomes and results of hypothesis assessment using a node classification scheme in yeast

Evolutionary outcome	Hypothesis (H)	Alternative Hypothesis (HA)	Results in this paper following assessment with hierarchical node classification scheme.
**Speed of evolution**	Indispensable or essential genes are more constrained and evolve slowly [43].	Functionally important and thus functionally constrained genes evolve slowly, independent of dispensability [23]. Highly expressed genes evolve slowest [15, 16].	**HA:** Functionally most constrained genes (H-nodes) have the lowest substitution ratios of all categories, and are most highly expressed, but have lower scores of evolutionary rewiring than P and I-nodes.
**Speed of evolution**	Central nodes have the highest number of edges; evolve very slowly because any change will lead to maladaptive pleiotropic effects - causing balancing selection through cost of complexity. [38], [37], [20], [36]	Intermediate nodes evolve fastest as their higher number of edges allows for evolution through rewiring [44, 45].	**HA:** Nodes with highest number of edges are intermediate to the network, evolve fast (high ɷ) and have a high score of rewiring (ɣ), indicating that the substitution rate of these genes may be associated with evolutionary rewiring events.
**Speed of evolution**	Nodes with a low number of edges evolve fastest due to higher degrees of freedom, which allows for genetic adaptations minimizing pleiotropic effects [46], [38]	–	**H:** Peripheral nodes evolve fast (high ɷ) and have a high score of rewiring (ɣ), indicating that the substitution rate of these genes may be associated with evolutionary rewiring events.
**Convergent evolution**	Nodes with a low number of edges should be the prime target of convergent evolution. Pleiotropic negative effects are expected to be low, and mutations in them can maximize adaptation [38].	Peripheral nodes have the highest degrees of freedom and thus divergence is more likely than convergence in them. Convergent evolution should instead be favored in nodes that allow for genetic variance, while having reduced degrees of freedom (I-nodes) (This contribution).	**HA**: 21 out of 26 nodes with convergent evolution demonstrated in yeasts were classified as I- nodes by DFA, and five as P nodes. ɷ and CAI were similar to I-nodes, but none of these 26 nodes showed evidence of evolutionary rewiring.
**Genic evolution**	Adaptations can be characterized (either causative or correlative for the speciation process) by any number of divergent genes within the genome, whereas other genes are not associated with adaptation [47].	Only the complete phenotype is selected, the genic component is less important [10].	**H:** Different clusters of functionally similar nodes experience either higher, lower than expected or neutral rates of evolution across five species of yeast [41]. Causation or correlation to the speciation process not testable with data.

**Table 3:** Discriminant function analysis summary to assign node categories H, I, P to nodes within dataset. Wilks’ Lambda: 0.0704 approx. F (6,2152) = 992.780 *p* < 0.001

	Wilks Lambda	Partial Lambda	F-remove 21,076	*p*-value	Toler.	1-Toler. (R-sqr.)
Neighborhood connectivity	0.137	0.514	507.835	< 0.001	0.988	0.012
Betweenness centrality	0.105	0.673	261.039	< 0.001	0.994	0.006
Average shortest path length	0.133	0.528	480.907	< 0.001	0.983	0.017

**Table 4:** List of yeast genes that were found to adapt to novel environments, and were additionally shown to evolve these adaptations convergently across populations or species of yeast. Node hierarchy categories after discriminant function analysis (DFA) are shown in the first column. P - peripheral nodes, I - intermediate nodes

DFA estimated Node hierarchy	Gene symbol	ORF ID	Reference
**I**	STE11	YLR362W	Lang et al., 2013 [49]
**I**	STE12	YHR084W	Lang et al., 2013 [49]
**I**	STE4	YOR212W	Lang et al., 2013 [49]
**P**	KRE6	YPR159W	Lang et al., 2013 [49]
**I**	SFL1	YOR140W	Lang et al., 2013 [49]
**I**	STE5	YDR103W	Lang et al., 2013 [49]
**P**	ANP1	YEL036C	Lang et al., 2013 [49]
**I**	GCN1	YGL195W	Lang et al., 2013 [49]
**I**	ERG5	YMR015C	Gerstein et al., 2012 [50]
**P**	ERG7	YHR072W	Gerstein et al., 2012 [50]
**I**	CNE1	YAL058W	Lang et al., 2013 [49]
**I**	GPB1	YOR371C	Lang et al., 2013 [49]
**P**	KEG1	YFR042W	Lang et al., 2013 [49]
**I**	KRE5	YOR336W	Lang et al., 2013 [49]
**I**	TOH1	YJL171C	Lang et al., 2013 [49]
**P**	SUL4	YBR294W	Gresham et al. 2008 [51]
**I**	GAL3	YDR009W	Hittinger et al., 2004 [52] Stern, 2013 [38]
**I**	GIN4	YDR507C	Gresham et al. 2008 [51]
**I**	PDR1	YGL013C	Anderson et al. 2003 [53]
**I**	SGF73	YGL066W	Gresham et al. 2008 [51]
**I**	SET4	YJL105W	Lang et al., 2013 [49]
**I**	SIR1	YKR101W	Gresham et al. 2008 [51]
**I**	ACE2	YLR131C	Lang et al., 2013 [49]
**I**	GAS1	YMR307W	Lang et al., 2013 [49]
**I**	WHI2	YOR043W	Lang et al., 2013 [49]
**I**	CKA2	YOR061W	Gresham et al. 2008 [51]

### Aim 2: genetic constraint and network architecture influencing evolution

**Fig. 5:**
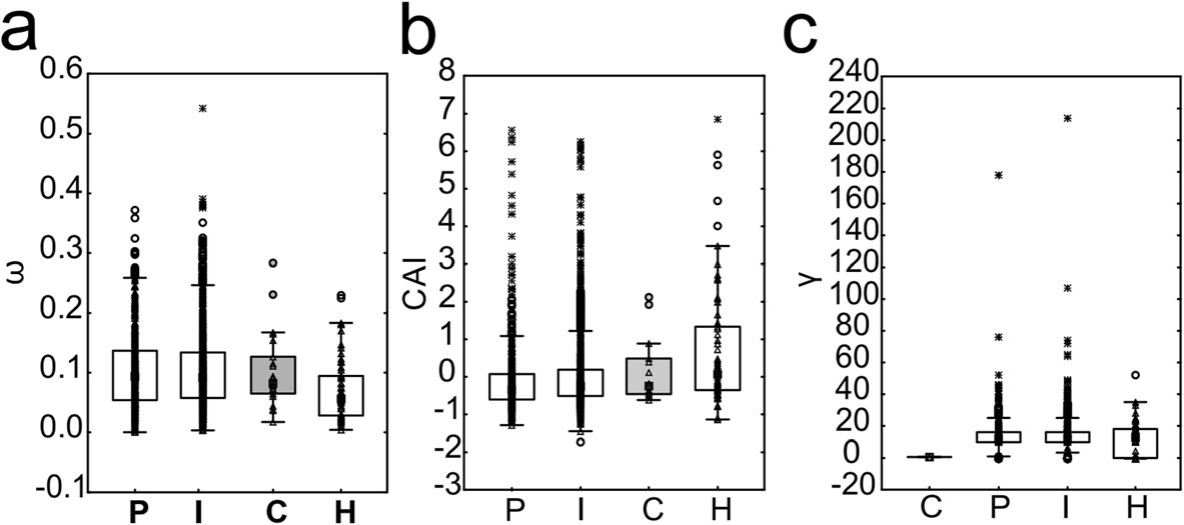
Relationship between hierarchical node structure of yeast interactome and evolutionary parameters. Node types are designated as peripheral (P), intermediate (I), or hub (H) based on discriminant function analysis, and nodes that were found to evolve convergently (C; *N* = 21) in yeasts. Three evolutionary outcomes (**a**) substitution rate, (**b**) expression level, approximated through Codon Adaptation Index (CAI), and (**c**) evolutionary rewiring score significantly differ among node categories (see text). C-node boxes are sorted by Median. Double red line: outliers above median not shown in figure but included in tests. Raw data points - triangles, circles - outliers, stars - extreme values, squares - Medians, boxes - 25-75% data, whiskers - non-outlier range

To test the influence of functional network constraint on the evolutionary outcomes rapid adaptation and convergent evolution, as well as gene expression, I rearranged and expanded on this [41] data set (see Methods). I then tested, how network statistical parameters relate to estimators of evolutionary parameters ω, γ, and CAI (Codon Adaptation Index). The amount of mRNA produced by each gene in regular somatic cells can be estimated by CAI which is derived from codon use bias in yeast that correlates with mRNA levels (based on [54, 55]). First, a general linear model was run with evolutionary parameters as dependent variables, and network parameters as predictor variables. All three network statistical parameters were found to significantly predict estimators for evolutionary outcomes (Table 5). All node categories have significantly different values for ω (KW-H(3,2204) = 20.1345, *p* = 0.0002), CAI (KW-H(3,2195) = 26.1472, *p* = 0.00001) and γ (KW-H(3,2195) = 36.7936, *p* = 0.00000), as shown by Kruskal-Wallis tests (Fig. 5). Figure 5 shows that the highest values of ω are found both in P and I-nodes with almost identical median values (0.93 vs. 0.91), while γ is highest in P and I-nodes and H nodes, as expected, had highest values of CAI.

**Table 5:** Multivariate Wilks tests of significance and powers for network parameters to explain protein evolutionary rate (ω), gene expression (Codon Adaptation Index CAI), and evolutionary rewiring between species of yeast (γ). All predictors were significant

	Wilks’ Lambda	F	Effect df	Error df	p	Observed power (alpha)
Intercept	0.317	1569.597	3	2188	< 0.001	1.000
Neighborhood connectivity	0.924	59.892	3	2188	< 0.001	1.000
Betweenness centrality	0.995	3.931	3	2188	0.008	0.832
Average shortest path length	0.961	29.553	3	2188	< 0.001	1.000

The effect size of network statistical parameters as predictors for variables estimating evolutionary outcomes relative to CAI was then determined. When CAI was incorporated into the analysis to predict values of ω and γ, average shortest path length (P-node classifier) was the predictor with highest power (0.99), followed by its interaction term with CAI (0.97), then CAI itself (0.92), followed by neighborhood connectivity (the I-node classifier, 0.83). Only betweenness centrality (the H-node classifier) was not significantly contributing to this model (Supplementary Tables 1 and 2). As mentioned previously, Fig. 5 shows that the estimator of gene expression levels is highest in H-nodes, which might explain why CAI was seen as a better predictor for ω and γ than betweenness centrality. However, AIC based model selection revealed that a global model of all four variables including CAI and network parameters explains ω and γ better than CAI itself (Supplementary Tables 3 and 4). For ω, the only model with higher likelihood than the global model is that excluding betweenness centrality, whilst the CAI-only model ranks 8th. The rewiring score γ is best explained by the global model and the CAI-only model ranks 5th.

## Discussion

### Aim 1: novel node classification scheme based on network statistics

One scenario of how functional genetic network architecture could influence evolutionary outcomes (Fig. 2) is the topography of nodes in a functional genomic network. When selection acts upon a population (for example, through a sudden change in climate), advantageous mutations will be selected from standing genetic variation (allele frequencies). A population will have standing genetic variation in different nodes of the network, which is dependent on the topological position of the nodes. Organisms possess a finite subset of biochemical pathways (underlying functional genetic networks) such as those related to temperature homeostasis [56–58], and that align to a finite amount of selected phenotype components. Figure 2 shows how the population must adapt to this newly arising selective pressure through selection of advantageous mutations in one of these subnetworks, but not by selecting mutations in any other subnetwork, as these are unrelated to the stimulus or organismal fitness in response to it, and would therefore not result in adaptation. This does not mean that other subnetworks are not under any selection, or under stabilizing selection for other causes, or that selection on one subnetworks does not influence others, but is a simplification here used for the purpose of classifying nodes. This constrains the number of mutations in the genome that selection will operate on, and thus determines the evolutionary response through genetic constraint. Second, and of high importance for the new classification scheme proposed here, node hierarchy within these subnetworks poses an additional level of constraint: and this additional level reduces the “evolutionary search space” for potential beneficial variants.

This can be illustrated through the following hypothetical construct, which reduces network structure to distinct types of nodes. Network nodes, which are functionally important for the operation of the network (hub nodes central to the network, H-nodes), are less likely to harbor significant genetic variation in first- or second-codon positions or regulatory regions because of their high functional constraint. Consequently, genetic variation, as well as adaptation to an environmental selective pressure, should both be more likely to occur within non-hub nodes within the subnetwork. Nodes with the highest number of edges are intermediately positioned within a network (intermediate nodes, I-nodes) and were shown to have weaker selective constraint [44, 45] than centrally positioned nodes, as they have lower functional constraint than H-nodes. Consequently, they should evolve faster. This assumption differs from the gene pleiotropy hypothesis, which places the highest functional constraint on these nodes (Table 2). However, because of their high cost of complexity, adaptation in I-nodes should be highly constrained in terms of which genes can adapt (depending on the nature of their functional interactions) and how (through changing the wiring pattern with other nodes). Because of gene pleiotropy, adaptations that do evolve in these nodes should have a larger phenotypic effect, which combined with the reduced possibilities for adaptation, increases the likelihood for convergent evolution in them. Genes peripheral in the network (peripheral nodes, P-nodes) have higher degrees of freedom due to the lowest degree of gene pleiotropy and should be able to accumulate genetic variation with least cost. Therefore, the population should already harbor more genetic variation within these peripheral genes on which selection can operate. Change in such nodes however, due to lower gene pleiotropic interactions, would result in less phenotypic effect and thus they are less likely to promote large evolutionary changes. In such nodes, divergence is more likely to accumulate than convergence. The expectation is thus that different node types will differ in standing genetic variation due to the different genetic constraints acting upon them. H-nodes will be very strongly constrained and only can accumulate little standing genetic variation, resulting in a low potential for selection to operate on. I-nodes will harbor sufficient standing genetic variation but be under high functional constraint, so that selection can only operate on a limited number of variants that all have multiple phenotypic effects. In different organisms, the same variants can be selected quickly due to this reduced search space, which leads to parallel genomic evolution resulting in convergent phenotypes.

P-nodes will be least constrained, allowing a lot of variation but less sweeping phenotypic effects due to lower gene pleiotropy. Selection can operate on multiple variants in these; selective advantages are more likely due to the lower gene pleiotropy in more genes, so selection will less likely lead to convergence. All three evolutionary outcomes can (among other factors such as gene expression levels) be explained with this mechanism of constraint through functional genetic network structure. As Fraser [59] pointed out, objective classification of nodes into any such type of categories is important, and they should reflect true topological properties of an interactome. In this study, significant support was found that the yeast network can be partitioned into these node categories, based on the network statistical parameters neighborhood connectivity, betweenness centrality, and average shortest path length.

Fraser’s [59] first exploration of the influence of modules within genomes and their hub nodes (called “modularity”) found that the rate of protein evolution is faster in intramodule hubs (nodes that link genes with high co-expression in response to a stimulus) compared to intermodule hubs (linking low genes with low co-expression in response to a stimulus, defined after [60]. The node classification scheme of Fraser [59] was based on gene co-expression and not on node topology, and gene co-expression in response to a stimulus followed a bimodal distribution. In this contribution, I nodes instead have the highest number of edges and connect sub-networks, but are not defined with respect to their expression (Table 2). In line with the expectations outlined above, C-nodes previously identified [38, 48–52] to have undergone convergent genomic adaptation in independent experiments, strains, or species of yeasts were located within the I-nodes category, showing that convergently adapting genes have similar evolutionary rates, expression levels, and degrees, as nodes that are located intermediately in the interactome.

### Aim 2: genetic constraint and network architecture influencing evolution

A recent paper published by Schoenrock et al. [41] uses a data set of 4179 protein-coding genes (sourced from 13,40) to investigate the involvement of network structure in protein evolution. This data set was generated for five species of yeasts (*Saccharomyces cerevisiae*, *S. paradoxus*, *S. bayanus*, *S. kudriavzevii*, and *S. mikatae*). The study compared a quantitative variable related to network structure (computationally predicted re-wiring of nodes through evolution γ), with an estimator of protein evolutionary rate on nodes (substitution rate ω, measured as dN/dS). The authors found that the degree of rewiring of nodes across the phylogeny was only poorly associated with evolutionary sequence divergence, but nodes with very low evolutionary rate had high variability of rewiring scores, which indicates that changing gene interactions is an important mechanism how functionally constrained genes may evolve. While the study remained somewhat inconclusive about the influence of network structure and node rewiring on protein evolution, the data contained within it, combined with additional data, allowed me to test the hypotheses outlined above using the new node classification scheme (Table 2). In this study, all three estimators of evolutionary parameters (substitution rate, re-wiring score, and gene expression levels) were significantly predicted by the three node categories.

With respect to rapid adaptation, the highest estimated substitution rates are found both in P and I-nodes, which shows that nodes located less centrally in the network evolve faster than other nodes. However, peripheral nodes were not identified as adapting particularly fast. CAI increases towards the center of the network, with mRNA expression level being highest in hub nodes. Network node hierarchy may therefore be able to explain the E-R anticorrelation (gene expression levels being negatively correlated with evolutionary rate [14]. H-nodes connect various subnetworks with one another, and thus are likely to be involved in more diverse functions (which might be partitioned across different tissues, processes or life history phases), than nodes more peripheral in a network (Figs. 5, 54). Such common functions may require a high amount of product, which may translate into high levels of mRNA expression in these nodes. γ is highest in P and I-nodes, indicating that evolutionary rewiring events are more common in less central parts of the networks. An interesting subject for further study may be to compare explicit topologies of nodes that underwent re-wiring through evolution, in order to determine whether they can additionally move between I and P node categories over time. I-nodes harbor the majority of edges within a network - genetically re-wiring these nodes could lead to rapid adaptation [61]. Centrality of H-nodes seems to reduce their adaptability while peripheral and intermediate nodes are less constrained to adapt, and this process may involve rewiring within the network. This demonstrates how functional constraint can explain evolutionary outcomes better than estimators for gene dispensability can. Rapid genomic adaptation within diversifying populations has been shown to occur as a rapid response to selection such as anthropogenic pollution [62]. Such rapid adaptation often occurs in the presence of gene flow [47, 62]. This means that adaptation is constrained to specific genes under selection, which can interrupt their gene flow between populations, while alleles of other genes show uninterrupted gene flow. The speed of such adaptation related to divergence of a subset of genes within the same genome has been dubbed the “genic theory of speciation” [47]. Such genic evolution was shown to occur in *Timema* stick insects [63]. Future studies could test whether such rapidly adapting loci are preferentially located in P- and I-nodes, and whether this leads to a change in inter-node wiring patterns.

With respect to genic adaptation, Schoenrock et al. [41] could show that some functionally similar nodes experienced lower than expected levels of protein evolution, indicating purifying selection. Nodes that were evolving through fewer re-wiring events than expected, included functions related to phosphorylation, mitochondrial translation, response to pheromone, small GTPase mediated signal transduction, and transport. Nodes that were evolving among the five yeast species with higher than expected degrees of re-wiring, included the functions metabolic process, and various gene ontologies related to transcription and its regulation, as well as the regulation of transposition regulation. As indicated in Fig. 2, these results prove that evolutionary outcomes are different for functionally different subnetworks within an interactome. It might be worth noting that, as outlined above, none of these functions is particularly related to growth but rather to maintaining organismal function, which is why they would be overlooked if conserved genes were only classified by the criterion of dispensability for colony growth. Gresham et al. [51] similarly showed that evolutionary constraint in experimentally evolved yeast populations over 200 generations is dependent on the type of selection (limiting Glucose or Phosphate vs. Sulphur), with convergence being an outcome of the system level organization of the respective metabolic pathway. Additionally, the same differences in evolutionary estimators between node categories that could promote rapid adaptation, would allow genes in different node categories to evolve with differential speed, which would allow for genic adaptation.

Traditionally, convergence has been studied in non-model organisms, and with a focus on adaptive modification of the phenotype (e.g., [64]). More recently, phenotypic convergence has been traced back to in some instances resulting from identical genotypic variants (called “genomic re-use”, reviewed in [38]). These can arise either as new parallel mutations or from parallel selection of the same alleles from standing genetic variation [38] such as in the independent selection of body armor in the ectodysplasin locus of stickleback fish [65]. Other examples have recently been uncovered in skin toxin transport in poison frogs, [66] or in functional genomic adaptation to cold in a range of extant and extinct mammals including the mammoth [67]. However, convergent phenotypic adaptations can alternatively be produced by different genes, and a recent study on convergently evolved *Anolis* lizard ecomorphs found no convergence at the amino acid level [68]. Convergence events may also be exaptations, where a similar allele evolved due to ancestrally different selective pressures with a subsequent change of function [69]. Genomic re-use in some distantly related lineages but not in others, may indicate that constraint at the genomic level limits the evolutionary search space, but can manifest in different ways at the nucleotide level. In this study, a small number of convergently adapting genes in yeasts were preferentially located within I-nodes, as aligned to this hypothesis. This supports the notion that nodes with the highest number of edges and intermediate network position are constrained to adapt and thus increase the likelihood for convergent evolution. Gresham et al. [51], from which five C-nodes were obtained, also showed that convergent evolution is related to system level organization of the respective metabolic pathway. In summary, these results clearly demonstrate a relationship between network architecture and convergence, and if additional genes will become known to evolve convergently in yeast, this hypothesis can be further tested.

Previous studies had identified gene expression level estimates (using CAI as proxy), not network topographical structure, as the predominant explanatory variable for functional genetic constraint influencing evolutionary outcomes. However, I could show here that in model ranking including CAI as an additional predictor for ω and γ, CAI in itself did not emerge as the best predictor. These results confirm that whilst gene expression levels are an important element of genetic constraint, the position of highly expressed nodes as hub nodes in the network, together with the other network topology parameters, yield better explanatory power for two estimators of evolutionary outcomes. These results further support network topology as an important agent of evolutionary constraint.

## Conclusions

Metagenomic resequencing of every 500 generations within a 60,000 generation *E. coli* long term evolution experiment [70] revealed that certain genes accumulated beneficial mutations through selection significantly more often than expected by chance, and were very often affected by parallel adaptation [70]. These results, together with the incidences of recurrent genomic adaptations reviewed herein, demonstrate that the above-described relationship between network structure and convergent evolution may be expandable to organisms other than yeasts [38]. Apart from the quick assessment performed in this contribution, the influence of network structure in shaping evolutionary outcomes in more complex organisms than yeast such as vertebrates still needs to be comprehensively tested. Additionally, statistics computed on edge distributions in non-model organisms may change over time as more experimental evidence on interactions becomes available, and evolutionary constraint might differ by the type of interactions studied.

As demonstrated above in the yeast example, the impending advent of large-scale functional genomic networks for many new species makes it possible to convert functional genomic network structure of related species into variables describing hierarchical node position within the network. Future tests relating evolution to genomic constraint could include node architecture, and revolve around (i) Comparing standing genetic variation to network node position (while considering the effect of demography, selective sweeps, genetic drift, bottlenecks, and other levels of extrinsic constraint); (ii) Testing whether similar subnetworks/node hierarchies adapt to same selection pressure in different organisms. (iii) Comparing the speed of realized adaptation to a mutation/selection expectation, without considering network constraint. The potential benefits of better understanding genetic constraint leading to deterministic evolution may be wide ranging-- in humans, the use of functional interaction networks is omnipresent in genomic and transcriptomic study of cancer data, and recently, calls have been made for evolutionary methods to be applied to cancer problems [71]. A recent study demonstrates how the early progression of pancreatic cancer is defined through evolutionary constraints resulting from following one of three tumor suppressive pathways, and thus may be predictable [72]. Recognizing network constraint as an evolutionary force, rather than as a juxtaposition of evolution through natural selection [73], would allow us to quantify “background genetic constraint” through functional network structure. The remaining variance could then be better allocated to mutation and selection in directing rapid, convergent, and genic phenotypic evolution.

## Methods

To assess evolutionary outcomes rapid adaptation and convergent evolution, as well as to address the important factor of gene expression in shaping protein-coding gene evolution, the data set of Schoenrock et al. including yeast ORF ID, computationally predicted evolutionary PPI re-wiring score (γ), and substitution rate (ω) [41] was downloaded. The re-wiring score was obtained from comparing networks across five species of yeast [41] and was used here to assess whether nodes that change wiring patterns are linked to specific positions within the network. The dataset was then rearranged and integrated with data downloaded from Wall et al. [15] including ORF ID, and CAI (Codon Adaptation Index, a measure of RNA expression levels, based on [54]. When analyzing networks, it is important to do so on exhaustive data sets [74] to avoid experimental bias [48]. Such an exhaustive interactome for yeast generated from the BIOGRID database [75] was obtained from CYTOSCAPE v.3.6.0 [76], which contained 6508 nodes and 340,000 edges, with data curated from 5500 studies. With the goal to calculate a classifier that will aid in describing hierarchical node position within networks, common network statistical parameters were calculated from this exhaustive yeast interactome in CYTOSCAPE v.3.6.0 [76] using the Network Analyzer function. Data for the matching node ORFs were appended to the data set, and variables with non-normal distribution were BoxCox transformed. The final data set contained 2209 ORFs with only a few missing data points per variable. The network statistical parameters obtained from the yeast interactome were average shortest path length (maximal in peripheral nodes), neighborhood connectivity (maximal in nodes intermediate to the network), and betweenness centrality (maximal in nodes connecting subnetworks). Nodes with maximum values for each one of these three statistical parameters, and that were not overlapping with each other (1081 nodes, Fig. 2), were each assigned to a category: P (peripheral nodes), I (intermediate nodes) and H (hub nodes). To assign node categories to the remaining nodes in the network that may be harder to allocate visually, a discriminant function analysis (DFA) was employed in STATISTICA (V13, Tibco). All remaining nodes with significant statistical support could be associated to one of these three categories (Table 3). To explore the network position of nodes that have undergone convergent adaptation, ORF IDs that were demonstrated experimentally to show convergent genomic adaptation in independent experiments, strains, or species of yeasts (C-nodes) were identified from the literature (66, 74–78), Table 4). Out of the 26 obtained C-nodes, 21 nodes were allocated by DFA to the I-category, and five were allocated to the P-category. It was then tested how network statistical parameters relate to the evolutionary parameters ω, γ, and CAI. First, a general linear model was run with evolutionary parameters as dependent variables, and network parameters as predictor variables. Differences in, respectively, network statistical parameters or estimators for evolutionary parameters, and node categories were tested with Kruskal-Wallis tests. Because previous studies [17–19] have ascribed gene expression (here measured as CAI) an important role for constraining evolution, it is possible that whilst network statistical parameters do explain evolutionary parameters well, this effect could disappear once CAI itself is considered as a predictor for ω and γ. This assumption was therefore tested through (i) comparing power of predictors in another linear model, including network statistical parameters, CAI, as well as interaction terms as predictors and (ii) comparing Akaike information criteria of models generated from these variables and their interaction terms.

## Supplementary information

**Additional file 1: Supplementary Table 1.** Results for linear multiple regression whole model. Test of significance between sum of squares (SS) of whole model vs. SS of residual. Factors were: average shortest path length, neighborhood connectivity, betweenness centrality, and CAI expression level. Significant effects are given in italics. **Supplementary Table 2.** Linear regression multivariate Wilks tests of significance for predictors and their interactions, effect sizes, and powers. Abbreviations are: ASPL - average shortest path length, NC- neighborhood connectivity, BC- betweenness centrality, CAI: gene expression level. Effect df = 2; error df = 2177. Significant predictors are given in italics. **Supplementary Table 3.** Generalized linear model (Normal distribution, log link function) model building results for **ω** as dependent variable. Given are degrees of freedom df, Akaike Information Criterion AIC, delta AIC, Likelihood ratio Chi square test, and resulting error probability. Abbreviations are: ASPL - average shortest path length, NC- neighborhood connectivity, BC- betweenness centrality, CAI: gene expression level. Significant models are given in italics. The full (global) model is highlighted in red. The model with only CAI as predictor is highlighted in gray. **Supplementary Table 4.** Generalized linear model (Normal distribution, log link function) model building results for **γ** as dependent variable. Given are degrees of freedom df, Akaike Information Criterion AIC, delta AIC, Likelihood ratio Chi square test, and resulting error probability. Abbreviations are: ASPL - average shortest path length, NC- neighborhood connectivity, BC- betweenness centrality, CAI: gene expression level. Significant models are given in italics.The full (global) model is highlighted in red. The model with only CAI as predictor is highlighted in gray.

**Additional file 2.** Data file.

## Data Availability

The datasets generated and/or analysed during the current study are included in this published article and available as Supplementary Table 5.

## Abbreviations

CAI: Codon Adaptation Index
ORF: Open Reading Frame
P-nodes: Peripheral nodes
I-nodes: Intermediate nodes
H-nodes: Hub nodes
γ: Computationally predicted re-wiring of nodes through evolution
ω: Substitution rate measured as dN/dS
