# Supplementary material for: Aligning functional network constraint to evolutionary outcomes
Source: BMC Evol Biol. 2020 May 24;20:58. doi: 10.1186/s12862-020-01613-8 (PMC7245893; doi:10.1186/s12862-020-01613-8)
Supplement: Supplementary file 1 — Additional file 1: Supplementary Table 1. Results for linear multiple regression whole model. Test of significance between sum of squares (SS) of whole model vs. SS of residual. Factors were: average shortest path length, neighborhood connectivity, betweenness centrality, and CAI expression level. Significant effects are given in italics. Supplementary Table 2. Linear regression multivariate Wilks tests of significance for predictors and their interactions, effect sizes, and powers. Abbreviations are: ASPL - average shortest path length, NC- neighborhood connectivity, BC- betweenness centrality, CAI: gene expression level. Effect df = 2; error df = 2177. Significant predictors are given in italics. Supplementary Table 3. Generalized linear model (Normal distribution, log link function) model building results for ω as dependent variable. Given are degrees of freedom df, Akaike Information Criterion AIC, delta AIC, Likelihood ratio Chi square test, and resulting error probability. Abbreviations are: ASPL - average shortest path length, NC- neighborhood connectivity, BC- betweenness centrality, CAI: gene expression level. Significant models are given in italics. The full (global) model is highlighted in red. The model with only CAI as predictor is highlighted in gray. Supplementary Table 4. Generalized linear model (Normal distribution, log link function) model building results for γ as dependent variable. Given are degrees of freedom df, Akaike Information Criterion AIC, delta AIC, Likelihood ratio Chi square test, and resulting error probability. Abbreviations are: ASPL - average shortest path length, NC- neighborhood connectivity, BC- betweenness centrality, CAI: gene expression level. Significant models are given in italics.The full (global) model is highlighted in red. The model with only CAI as predictor is highlighted in gray. [file 12862_2020_1613_MOESM1_ESM.docx]

**Supplementary Material**

Aligning functional network constraint to evolutionary outcomes

Katharina C. Wollenberg Valero

Containing: **Supplementary Tables 1-4**

**Supplementary Table 1.** Results for linear multiple regression whole mode. Test of significance between sum of squares (SS) of whole model vs. SS of residual. Factors were: average shortest path length, neighborhood connectivity, betweenness centrality, and CAI expression level. Significant effects are given in italics.

| **Dependent Variables** | **Multiple R** | **Multiple R²** | **Adjusted R²** | **SS Model** | **df Model** | **MS Model** | **SS Residual** | **Df Residual** | **MS Residual** | **F** | **p** |
| --- | --- | --- | --- | --- | --- | --- | --- | --- | --- | --- | --- |
| *γ* | *0.152* | *0.023* | *0.016* | *4767.874* | *15* | *317.858* | *202278.371* | *2178* | *92.873* | *3.422* | *=8.5e^-5^* |
| *ω* | *0.427* | *0.182* | *0.176* | *1.561* | *15* | *0.104* | *7.018* | *2178* | *0.003* | *32.294* | *<0.0001* |

**Supplementary Table 2.** Linear regression multivariate Wilks tests of significance for predictors and their interactions, effect sizes, and powers. Abbreviations are: ASPL - average shortest path length, NC- neighborhood connectivity, BC- betweenness centrality, CAI: gene expression level. Effect df = 2; error df = 2177. Significant predictors are given in italics.

| **Predictor variables and interaction terms** | **F** | **P =** | **Observed power (alpha=0.05)** |
| --- | --- | --- | --- |
| *Intercept* | *8.157* | *2.95e^-3^* | *0.960* |
| *{2} ASPL* | *11.643* | *9.34.e^-5^* | *0.994* |
| *ASPL*CAI* | *9.317* | *9.35e^-4^* | *0.978* |
| *{4} CAI* | *6.891* | *0.001* | *0.924* |
| *{1} NC* | *5.282* | *0.005* | *0.837* |
| *NC*ASPL* | *3.853* | *0.021* | *0.700* |
| {3} BC | 0.127 | 0.881 | 0.070 |
| NC*BC | 1.194 | 0.303 | 0.262 |
| ASPL*BC | 0.123 | 0.884 | 0.069 |
| NC*CAI | 4.799 | 0.008 | 0.798 |
| BC*CAI | 0.167 | 0.846 | 0.076 |
| NC*ASPL*BC | 1.227 | 0.294 | 0.269 |
| NC*ASPL*CAI | 3.549 | 0.029 | 0.661 |
| NC*BC*CAI | 1.183 | 0.306 | 0.260 |
| ASPL*BC*CAI | 0.160 | 0.852 | 0.075 |
| 1*2*3*4 | 1.216 | 0.297 | 0.267 |

**Supplementary Table 3.** Generalized linear model (Normal distribution, log link function) model building results for **ω** as dependent variable. Given are degrees of freedom df, Akaike Information Criterion AIC, delta AIC, Likelihood ratio Chi square test, and resulting error probability. Abbreviations are: ASPL - average shortest path length, NC- neighborhood connectivity, BC- betweenness centrality, CAI: gene expression level. Significant models are given in italics. The full (global) model is highlighted in red. The model with only CAI as predictor is highlighted in gray.

| **Model No.** | **Var. 1** | **Var. 2** | **Var. 3** | **Var. 4** | **df** | **AIC** | **dAIC** | **L.ratio Chi^2^** | **p** |
| --- | --- | --- | --- | --- | --- | --- | --- | --- | --- |
| *1* | *CAI* | *ASPL* | *NC* |  | *3* | *-6447.116* | *--* | *519.516* | *<1e^-8^* |
| *2* | *CAI* | *ASPL* | *BC* | *NC* | *4* | *-6446.727* | *0.389* | *521.126* | *<1e^-8^* |
| *3* | *CAI* | *BC* | *NC* |  | *3* | *-6427.143* | *19.584* | *499.541* | *<1e^-8^* |
| *4* | *CAI* | *NC* |  |  | *2* | *-6424.137* | *3.006* | *494.536* | *<1e^-8^* |
| *5* | *CAI* | *ASPL* | *BC* |  | *3* | *-6390.305* | *33.832* | *462.704* | *<1e^-8^* |
| *6* | *CAI* | *BC* |  |  | *2* | *-6389.946* | *0.358* | *460.345* | *<1e^-8^* |
| *7* | *CAI* | *ASPL* |  |  | *2* | *-6381.948* | *7.998* | *452.347* | *<1e^-8^* |
| *8* | *CAI* |  |  |  | *1* | *-6379.731* | *4.218* | *448.130* | *<1e^-8^* |
| *9* | *ASPL* | *BC* | *NC* |  | *3* | *-6065.065* | *173.203* | *137.463* | *<1e^-8^* |
| *10* | *ASPL* | *NC* |  |  | *2* | *-6064.246* | *2.819* | *134.645* | *<1e^-8^* |
| *11* | *BC* | *NC* |  |  | *2* | *-6016.366* | *47.880* | *86.764* | *<1e^-8^* |
| *12* | *NC* |  |  |  | *1* | *-6008.095* | *8.271* | *76.494* | *<1e^-8^* |
| *13* | *ASPL* | *BC* |  |  | *2* | *-5967.606* | *40.479* | *38.005* | *=5.58e^-8^* |
| *14* | *BC* |  |  |  | *1* | *-5957.836* | *9.770* | *26.235* | *=3.02e^-6^* |
| *15* | *ASPL* |  |  |  | *1* | *-5950.705* | *7.131* | *19.104* | *=1.24e^-4^* |

**Supplementary Table 4.** Generalized linear model (Normal distribution, log link function) model building results for **γ** as dependent variable. Given are degrees of freedom df, Akaike Information Criterion AIC, delta AIC, Likelihood ratio Chi square test, and resulting error probability. Abbreviations are: ASPL - average shortest path length, NC- neighborhood connectivity, BC- betweenness centrality, CAI: gene expression level. Significant models are given in italics.The full (global) model is highlighted in red. The model with only CAI as predictor is highlighted in gray.

|  | **Var. 1** | **Var. 2** | **Var. 3** | **Var. 4** | **df** | **AIC** | **dAIC** | **L.ratio Ch^i^2** | **p** |
| --- | --- | --- | --- | --- | --- | --- | --- | --- | --- |
| *1* | *CAI* | *ASPL* | *BC* | *NC* | *4* | *16175.287* | *--* | *39.607* | *5.21e^-7^* |
| *2* | *CAI* | *ASPL* | *NC* |  | *3* | *16176.924* | *1.637* | *35.969* | *7.6e^-7^* |
| *3* | *CAI* | *BC* | *NC* |  | *3* | *16177.088* | *0.164* | *35.806* | *8.23e^-7^* |
| *4* | *CAI* | *NC* |  |  | *2* | *16177.386* | *0.298* | *33.508* | *5.29^e-7^* |
| *5* | *CAI* |  |  |  | *1* | *16189.137* | *11.751* | *19.757* | *8.79e^-5^* |
| *6* | *CAI* | *BC* |  |  | *2* | *16190.230* | *1.093* | *20.664* | *3.25e-4* |
| *7* | *CAI* | *ASPL* |  |  | *2* | *16191.136* | *0.906* | *19.757* | *5.12e-4* |
| *8* | *CAI* | *ASPL* | *BC* |  | *3* | *16192.222* | *1.085* | *20.672* | *0.000123* |
| *9* | *BC* | *NC* |  |  | *2* | *16198.187* | *5.965* | *12.707* | *0.00174* |
| *10* | *ASPL* | *BC* | *NC* |  | *3* | *16199.130* | *0.943* | *13.764* | *0.00324* |
| *11* | *NC* |  |  |  | *1* | *16200.573* | *1.443* | *8.320* | *0.00392* |
| *12* | *ASPL* | *NC* |  |  | *2* | *16202.332* | *1.759* | *8.562* | *0.0138* |
| 13 | BC |  |  |  | 1 | 16206.448 | 4.116 | 2.446 | 0.118 |
| 14 | ASPL | BC |  |  | 2 | 16208.332 | 0.128 | 2.562 | 0.278 |
| 15 | ASPL |  |  |  | 1 | 16208.584 | 0.252 | 0.310 | 0.578 |
